# Rare case of extra-peritoneal brown fat (hibernoma) mimicking dumbbell-shaped liposarcoma

**DOI:** 10.1259/bjrcr.20200162

**Published:** 2021-05-12

**Authors:** Alex Kiu, Tiffany Fung, Sungmi Jung, Rehana Jaffer, Marie-Helen Martin

**Affiliations:** 1 Department of Diagnostic Radiology, McGill University Health Center, MontrealQC, Canada; 2 Department of Pathology, McGill University Health Center, MontrealQC, Canada

## Abstract

Hibernomas are a very rare and benign soft tissue tumour that originate from brown adipose tissue. While they are not histologically malignant, they may be indistinguishable from aggressive tumours such as liposarcomas on imaging. It is, therefore, important to consider it as a differential diagnosis when a suspicious fatty lesion is seen on imaging. This may prevent unnecessary invasive surgery and patient stress. This paper illustrates the clinical presentation, radiological features, and histological diagnosis of a patient with a rare dumbbell-shaped hibernoma in the pelvis.

## Case presentation and image findings

**Figure 1.**
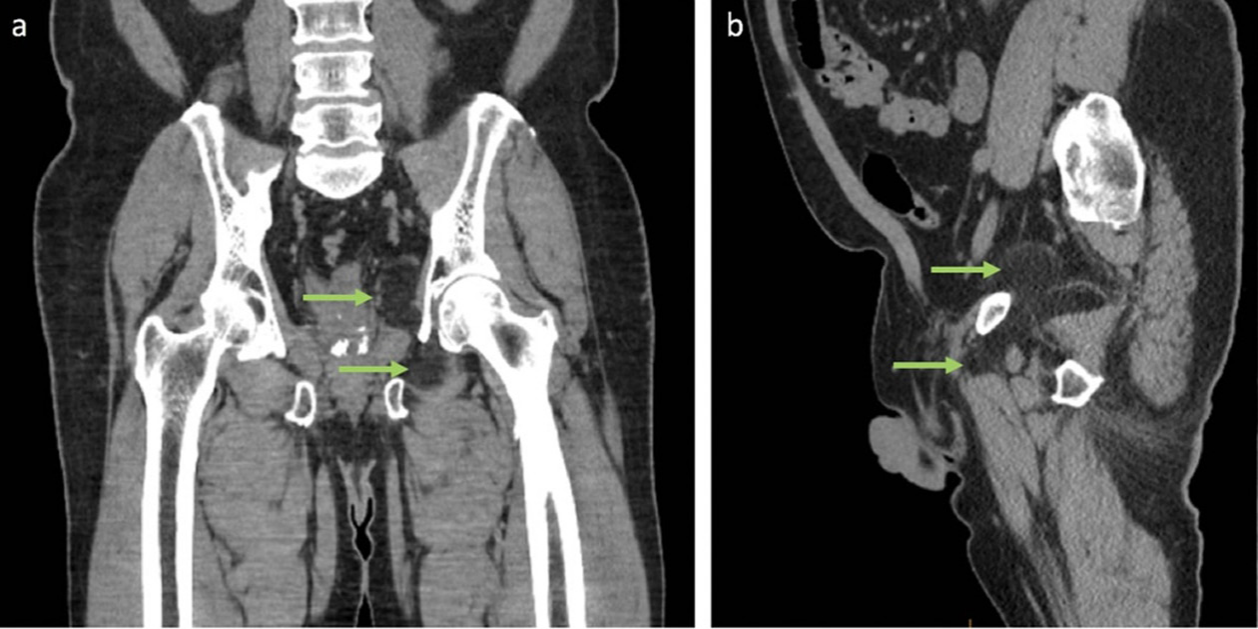
(a – b) Computed tomography (CT) images of the pelvis with coronal and sagittal views showing a bilobed, dumbbell-shaped fat density lesion in the left obturator canal (green arrows).

**Figure 2.**
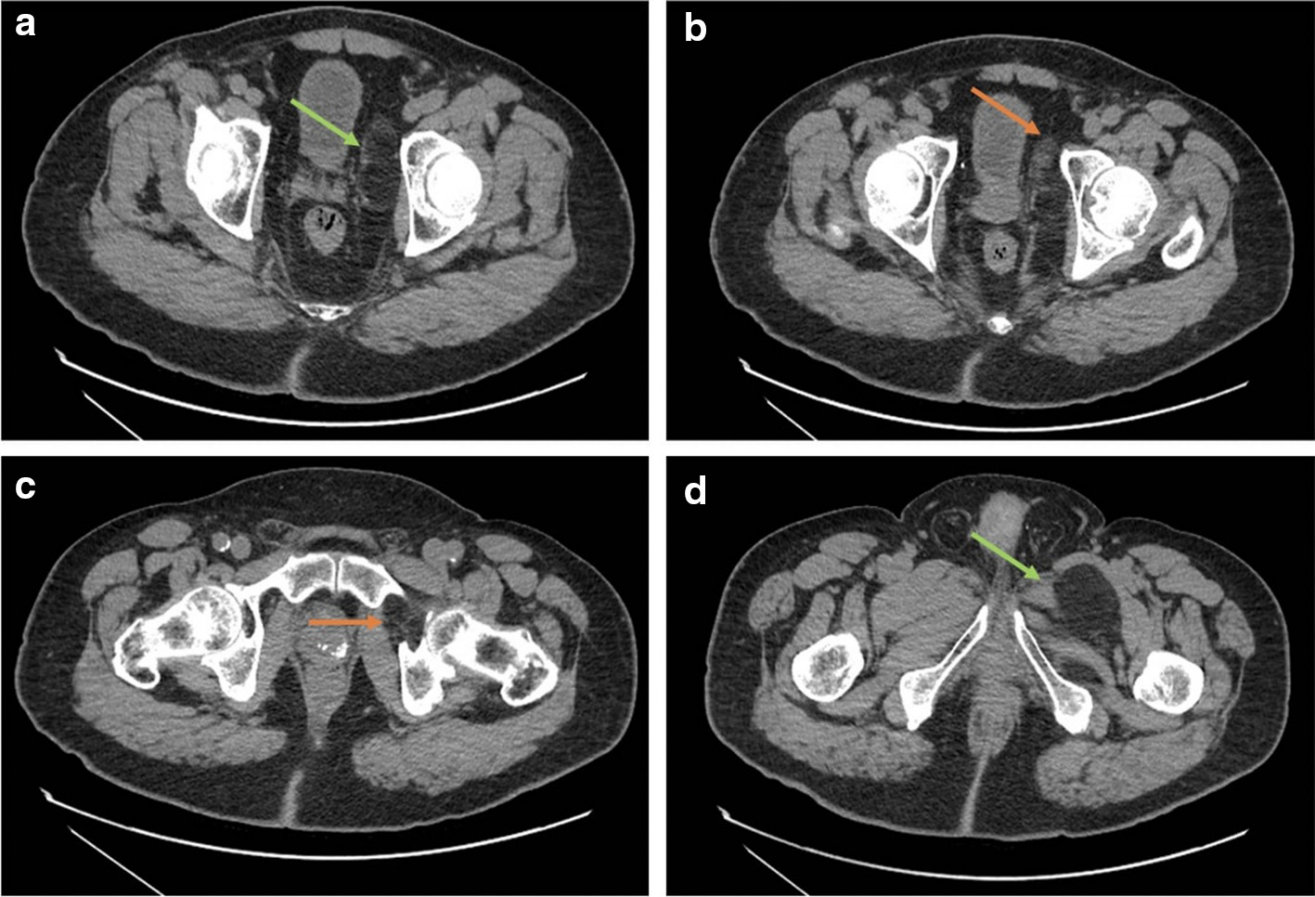
(a – d) CT images with axial views demonstrating the fat density lesion extending from the obturator canal into the left thigh. Green arrows demonstrating normal fat. Orange arrows demonstrating abnormal aggressive features. 2b**.** A solid soft tissue nodule is seen within the lesion. 2c**.** Septations areseen within the lesion.

**Figure 3.**
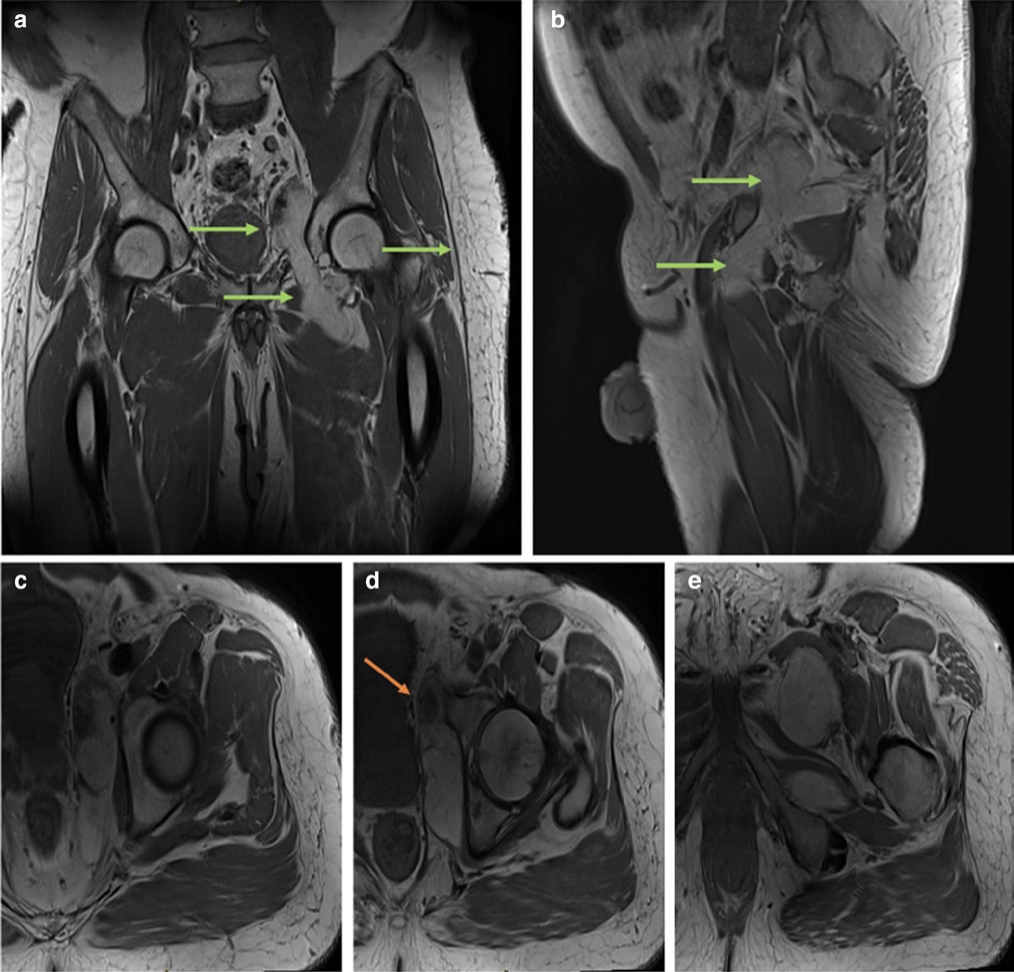
(a – e) Magnetic resonance imaging (MRI): Corresponding coronal, sagittal and axial *T_1_*-weighted sequences demonstrating bright fatty signal within the lesion (note of same signal with the subcutaneous fat). Green arrows demonstrating normal fat. Orange arrows demonstrating abnormal aggressive features. 3d. Solid nodule (low signal compared to the fat) within the lesion.

**Figure 4.**
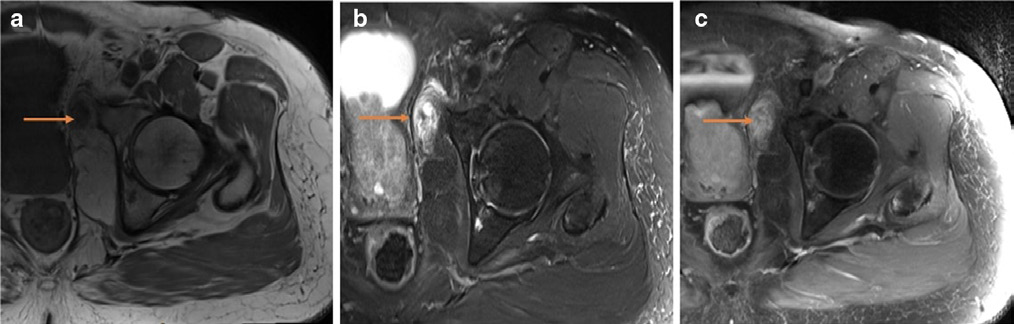
(a – c) MRI: (**a**) Axial *T_1_*-weighted, (**b**) fat suppressed *T_2_*-weighted and (**c**) fat suppressed *T_1_*-weighted with contrast demonstrating solid nodule that is low T1, high T2 with solid heterogeneous enhancement (orange arrow); imaging features supportive of an aggressive lesion. Orange arrows demonstrating abnormal aggressive features.

**Figure 5.**
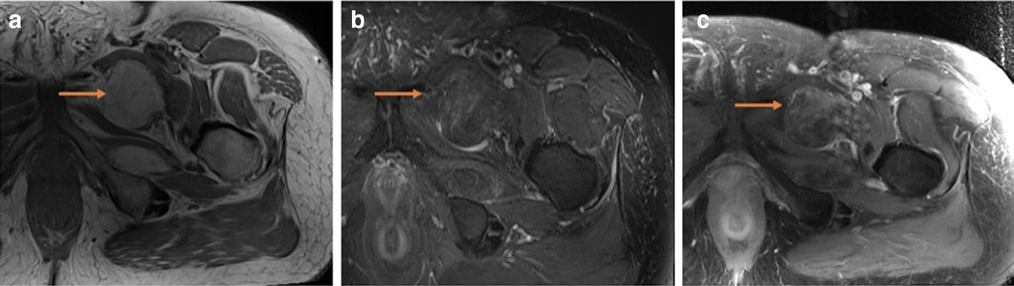
(a – c) (**a**) Axial *T_1_*-weighted, (**b**) fat suppressed *T_2_*-weighted and (**c**) fat suppressed *T_1_*-weighted with contrast demonstrate mild internal septations with high T2 signal and heterogeneous enhancement (orange arrow): imaging features supportive of an aggressive lesion. Orange arrows demonstrating abnormal aggressive features.

**Figure 6.**
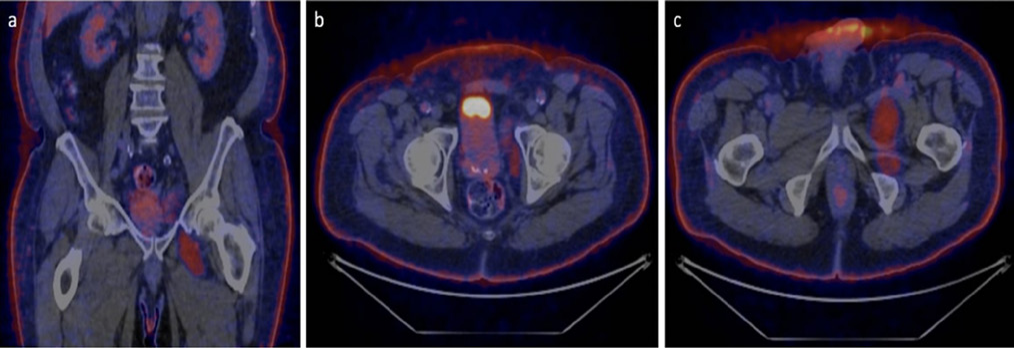
(a – c) PETCT (Positron emission tomography FDG with CT) demonstrating increased uptake within the entire lesion when compared with the background normal fat (no uptake). SUV of the lesion including the enhancing nodule/septation measured 6.0–7.8. (Lesions within SUVs of 2.0 are considered benign).

**Figure 7.**
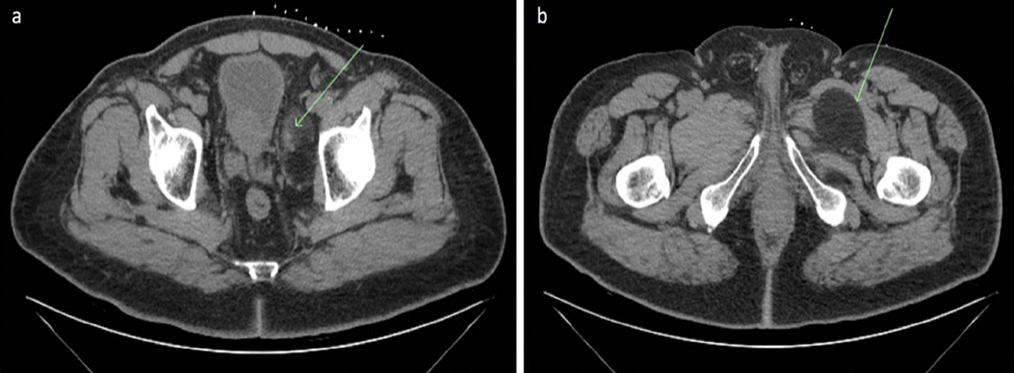
(a – b) Axial CT images of the biopsy tract targeted at the area of enhancement within the upper soft tissue nodule and the septations within the lower border of the lesion.

**Figure 8.**
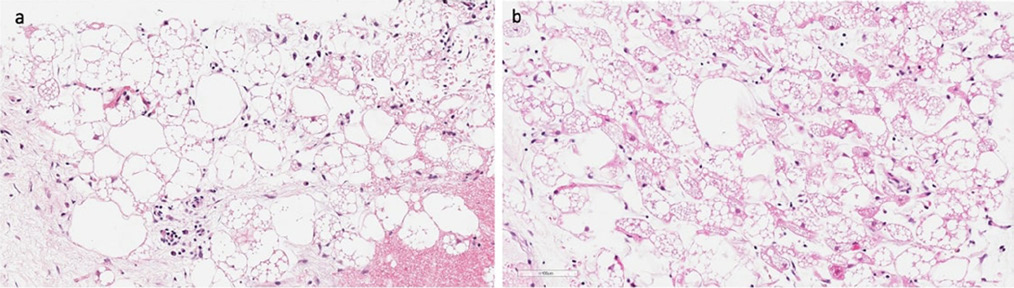
(a – b) Histopathology. 8a. Composed mostly of brown fat cells and occasional mixed white fat cells. (Corresponds with Figure 7a) 8b. Brown fat cells with eosinophilia and granular multivacuolated cytoplasm and centrally located small nucleus. (Corresponds with Figure 7b)

A 71-year-old male, who was undergoing investigations for kidney disease, was found to have an incidental left-sided lipomatous lesion extending from his pelvic sidewall through the left obturator canal on contrast-enhanced CT (Figures 1–2). An MRI was ordered for further characterization which revealed a bilobed, dumbbell-shaped lobulated lipomatous mass with both intra- and extra pelvic components (Figures 3–6). The lesion measured 12.2 × 8.1 × 4.1 cm with irregular, heterogeneous enhancement concerning for a liposarcoma. An FDG-PET was ordered which demonstrated hypermetabolic activity in the region of interest (Figure 7). A CT-guided core biopsy was performed, and the specimen was sent to pathology which confirmed a hibernoma (Figure 8).

## Differential diagnosis

The differential diagnosis for a fatty mass would include but not exhaustive of a lipoma, liposarcoma, myolipoma or hibernoma. The findings on CT and MRI of septations and solid nodule enhancement favour an aggressive lesion, for example, liposarcoma over a hibernoma. The FDG-PETCT demonstrating increased uptake within the lesion suggests possible hibernoma or liposarcoma. On the basis of metabolic activity, hypermetabolic hibernoma cannot be differentiated with low-grade liposarcoma as the metabolic activity of a hibernoma is very variable and changes from time to time. To aid in diagnosis, diazepam or a β-blocker can be injected before the FDG-PETCT and if the metabolic activity of the lesion is suppressed, it would be compatible with a diagnosis of hibernoma. This method was not chosen in our study as a CT-guided core biopsy was preferred by the surgical team for a definite diagnosis. A biopsy was obtained with an 18-gauge Bio-Pince needle through a 17-gauge coaxial needle for histopathology assessment.

## Treatment and follow-up

In view of the histologically proven non-aggressive hibernoma, the soft tissue sarcoma surgical team was consulted with the patient ultimately deciding to be followed-up with annual imaging.

Repeat imaging of the mass one year later revealed no significant change.

## Discussion

Hibernomas are a rare entity of benign slow-growing soft tissue tumours. They were first described by Merkel in 1906 as a pseudolipoma of the breast^1^ and later by Gery in 1914 as a hibernoma due to its resemblance to brown fat in hibernating animals^2^ with only a few cases being reported in the literature since.^3^ They usually occur in young adults and are slightly more frequent in females.^4^ Hibernomas represent approximately 1.6% of benign lipomatous tumours with the more common sites being the thigh, shoulder or neck. However, we present a very rare case of a large, dumbbell-shaped hibernoma located in the pelvis.

Hibernomas are often asymptomatic and are often discovered incidentally or when patients present with a painless soft mass.^5^ However, hibernomas can grow and compress adjacent structures resulting in pain leading patients to seek medical attention,^3^ mimicking a more aggressive lesion. Radiological imaging would assist in characterisation of the lesion.

On imaging, ultrasound usually demonstrates a hyperechoic mass with feeding surface vessels on doppler imaging.^6^ On CT, hibernomas appear as a well-circumscribed mass with lobulated features and multiple internal but smooth septations.^6^ Typically, the heterogeneous mass has imaging features varying between muscle and subcutaneous fat.^7^ For more detailed characterization, MRI demonstrates an encapsulated mass with an intermediate signal intensity between that of subcutaneous fat and muscle. On MRI fat suppression images, the heterogeneous nature of the lipid mass and its linear septations may result in incomplete fat suppression.^4,7^ Further investigation with scintigraphy FDG-PET scans show an increased uptake of the lesion due to its high metabolic activity of glucose.^4^

These lesions often mimic malignant soft tissue tumours such as liposarcomas and thorough investigation is necessary to distinguish the two. Both hibernoma and liposarcoma may demonstrate internal heterogeneity, thick septa and contrast enhancement as in our case, and may also be similar in appearance of sharply defined borders or even with a pseudocapsule in cases of low-grade tumours (*e.g.,* low grade myxoid liposarcomas).^8^ There are four histological variants of hibernomas with the typical pathology demonstrating adipocytes and brown fat cells with granular eosinophilic cytoplasm.^5^ There are also four subtypes of liposarcomas: well differentiated, dedifferentiated, myxoid and pleomorphic.^8^

Hibernomas, in particular pelvic hibernomas as in our case are rare. Physicians should be aware of and consider them in the differential diagnoses of fatty soft tissue tumours as they have a very different management and prognosis compared to their more malignant counterpart. Radiological findings of hibernomas can mimic aggressive lesions such as liposarcomas and, therefore, soft tissue sampling is encouraged for definitive diagnosis. Imaging guided biopsy can help with this, potentially avoiding major surgery. The prognosis for hibernomas is very favourable, resulting in complete cure if surgically excised and no documented cases of malignant transformation or metastases.^5^

## Conclusion

This case report illustrates a very rare case of a pelvic hibernoma and highlights the importance of considering hibernomas in the differential diagnoses of aggressive appearing lipomatous lesions (liposarcomas) as this would dictate management. Appreciating this entity and difference may lead to prevention of major surgery.

## Learning points

1. Importance of including hibernomas in differential diagnosis of lipomatous soft tissue tumours as they can be radiologically indistinguishable from more aggressive/malignant lesions.
2. Importance of multimodality imaging in obtaining and narrowing a diagnosis. In this case report, the use of CT, MRI and FDG-PET helped narrow the differential.
3. Ultimately, however, a CT-guided biopsy with histopathological analysis provides definite diagnosis.
